# Topical Chlorhexidine‐Induced Oral Allergic Reaction: A Case Report

**DOI:** 10.1155/carm/2841092

**Published:** 2026-04-12

**Authors:** Matteo Zotti, Katia Rupel, Marina Diolosà, Giulia Ottaviani

**Affiliations:** ^1^ Department of Medicine, Surgery and Health Sciences, University of Trieste, Trieste, Italy, units.it; ^2^ Azienda Sanitaria Universitaria Giuliano Isontina, Trieste, Italy

**Keywords:** allergy, case report, chlorhexidine, hypersensitivity

## Abstract

This case report describes a rare episode of allergic reaction to the topical application of chlorhexidine gel. The patient, following the prescription from her private dentist, presented to the dental emergency unit, referring to the onset of an increasing swelling of the upper lip a few hours after the application of chlorhexidine gel. After the exclusion of a possible infective process starting from the maxillary incisors, the patient was prescribed prednisone and exhibited a quick remission of the swelling. This report aims to emphasize the importance of a correct differential diagnosis to warrant an optimal therapeutic approach to the patient, since swellings of the upper lip could be related to different conditions.

## 1. Introduction

Chlorhexidine is a widely used antiseptic in the medical field, since it is recommended for the management of several conditions, both surgical and nonsurgical. Among the nonsurgical ones, chlorhexidine has gained use in the dental field as it is contained in many toothpastes or gels for topical use, and it is suggested by the dental operators due to its antimicrobial properties. In particular, chlorhexidine finds its major use in the periodontal field, due to its proven effects on periodontal health [1–5]. However, allergic reactions represent a relevant problem in medicine. Among the wide spectrum of clinical presentations, angioedema is one of the possible manifestations of an allergic reaction. Often, it represents an episode of immediate hypersensitivity (Type I, according to Gell and Coombs), mediated by IgE. Clinical signs of such allergic reactions are a rapid onset time, leading to swelling often associated with itching, redness, and tingling. Furthermore, other signs and symptoms may be visible, such as urticaria, respiratory difficulty, and a sensation of throat tightness. This may represent life‐threatening conditions, and therefore, immediate medical management is mandatory.

## 2. Case Presentation

**FIGURE 1:**
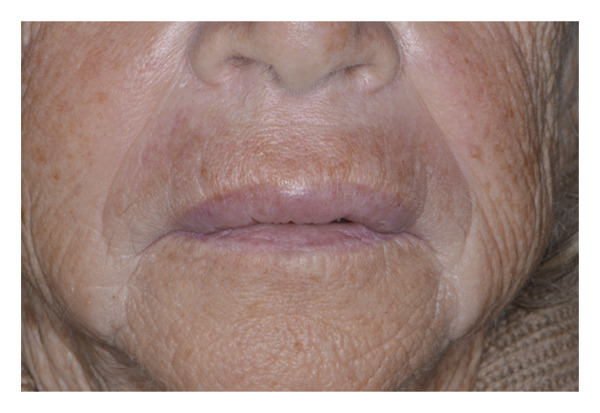
Initial extraoral photograph: Swelling of the upper lip is observable.

**FIGURE 2:**
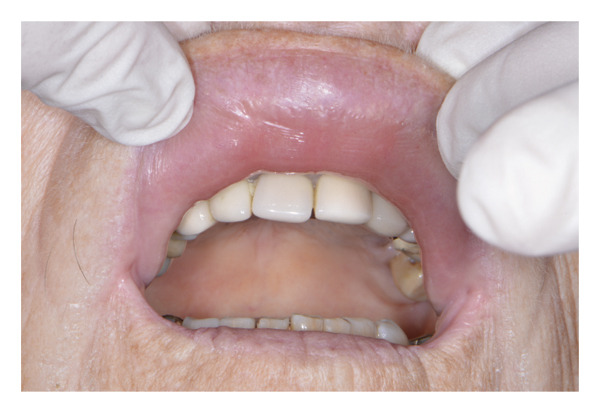
Initial intraoral photograph: Swelling of the upper lip is observable.

**FIGURE 3:**
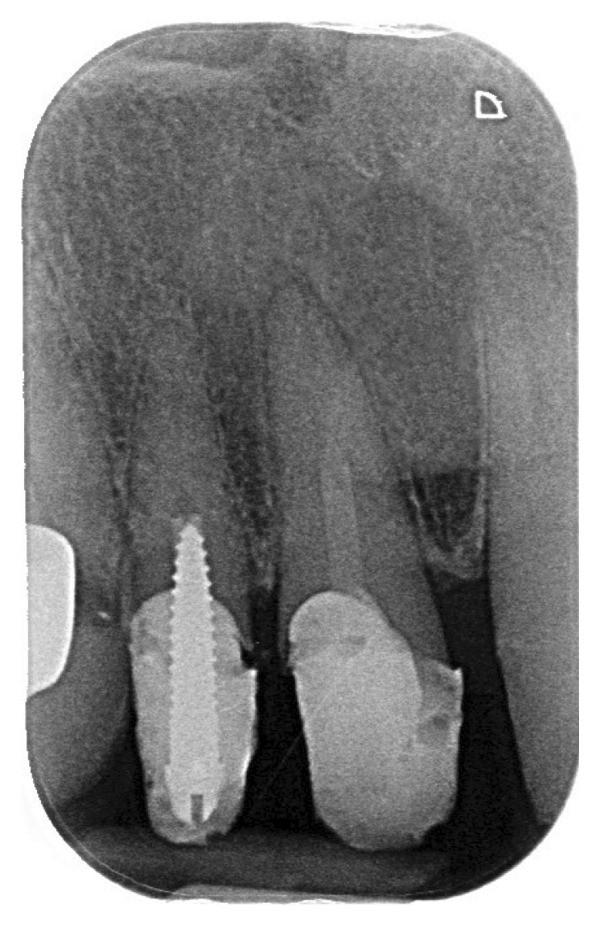
Intraoral radiograph of the central incisors: No periapical radiolucencies are observed.

**FIGURE 4:**
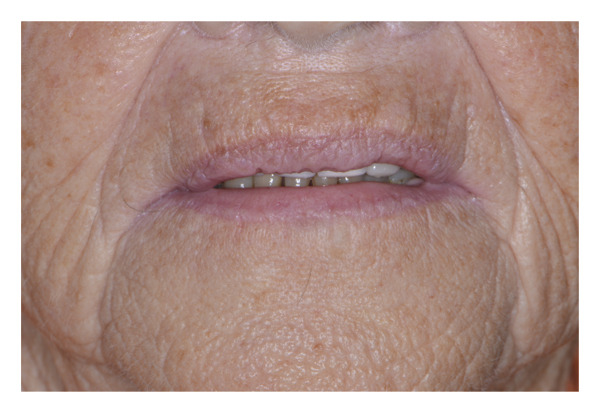
Extraoral photograph at 3‐day follow‐up: No swelling is observed.

**FIGURE 5:**
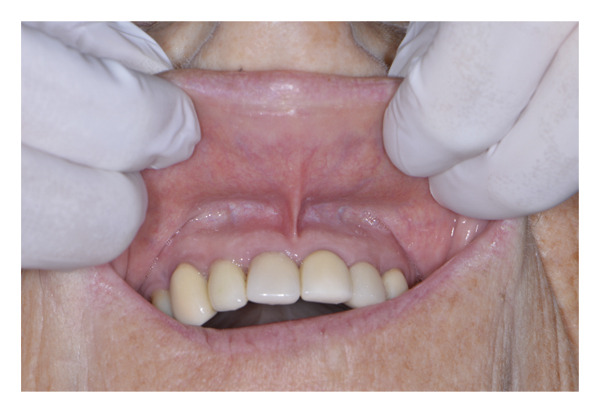
Intraoral photograph at 3‐day follow‐up: No swelling is observed.

An 87‐year‐old female patient presented to the dental emergency unit, reporting the onset of swelling of the mid‐third of the face the previous night. Her anamnestic history accounted for anticoagulant (rivaroxaban), due to valvular heart disease, and antiresorptive therapy (alendronic acid), due to osteoporosis. As a result of a recent diagnosis of gingivitis surrounding her frontal maxillary elements, the previous day, she was prescribed topical chlorhexidine (Curasept) by her private dentist. A few hours after its use, the patient reported the onset of swelling of the upper lip and therefore decided to take a tablet of paracetamol 1000 mg, without any improvement of the symptoms. The worsening of the swelling led her to present to the emergency unit in the following morning. On the extraoral examination, the patient presented a swelling of the upper lip with a flattening of the nasolabial fold, together with a light swelling of the right malar region (Figures 1 and 2). The intraoral examination revealed the presence of a poor oral hygiene condition, with the presence of many tooth decays and periodontal compromise. An intraoral X‐ray of the frontal incisors was performed, and no signs of periapical radiolucencies were noticed (Figure 3). Neither intraoral fistulae nor intrasulcular purulent drainage was noticed, as well as any swelling of the maxillary vestibulum was evident. Following informed consent, intra‐ and extraoral photographs of the patient were taken, with the aim of comparing the clinical evolution during the follow‐up. In this clinical case, the main differential diagnosis involved dental infections. Indeed, the presence of multiple chronic infective foci in the oral cavity could represent a trigger for its onset. However, the absence of a swelling of the maxillary vestibulum, together with the absence of active purulent discharge from the periodontal pocket, facilitated the exclusion of this condition. Furthermore, the intraoral X‐ray displayed any radiolucencies at the frontal maxillary teeth. Moreover, a dental abscess involving the maxillary anterior teeth could easily lead to a similar clinical aspect, with a swelling of the upper lip. The main key point for the differential diagnosis was the temporal link between the topical application of chlorhexidine and the rapid onset of the edema. Other possible conditions that may lead to a swelling of the upper lip may be due to trauma or autoimmune diseases (i.e., SLE or Melkersson−Rosenthal syndrome), which were soon excluded in the diagnostic process after an accurate medical history assessment. A systemic corticosteroid therapy was promptly initiated, and the patient has been monitored for 1 hour following the first administration. Specifically, prednisone 25 mg 1 tablet a day for 4 days, together with paracetamol 1000 mg in case of pain, maximum 3 tablets per day. The patient was visited 3 days later, and a complete resolution of the clinical signs was perceived (Figures 4 and 5). The patient was advised to consult her private dentist for the management of the dental condition.

## 3. Discussion

The international literature reports only a few cases of allergic reaction related to chlorhexidine use, in particular in the dental field. Mostly, chlorhexidine reactions developed after the exposure to extraoral surgical and nonsurgical devices, evolving as an urticarial‐like reaction [6, 7]. A recent review published by Solderer and Schmidlin showed that there is no evidence of allergic reactions after the use of chlorhexidine rinses [8]. In a case report by Kotsailidi et al., a delayed‐type of hypersensitivity reaction to chlorhexidine gel was described, presenting as an intense burning of the gingiva [9]. In the literature, two cases of life‐ending anaphylactic reactions have been described related to the administration of chlorhexidine for the irrigation of dental sockets, but they seem to represent a particularly rare occurrence [10]. Beyond the rarity of the allergic event itself, this case highlights a critical clinical intersection: the “masquerade” effect of chlorhexidine‐induced angioedema. When involving the upper lip, it perfectly mimics the clinical onset of an acute vestibular abscess. The originality of our report lies in demonstrating how a meticulous differential diagnosis is not merely an academic exercise but a fundamental safety measure for complex patients. Given the patient’s pharmacological regimen, including rivaroxaban and alendronic acid, a misdiagnosis could have led to unnecessary and high‐risk invasive procedures, such as emergency drainage or extraction, exposing the elderly patient to potential iatrogenic complications such as uncontrolled bleeding or medication‐related osteonecrosis of the jaw (MRONJ). In this study, the critical importance of a thorough differential diagnosis is highlighted. This process is foundational to providing effective and safe medical care. The cornerstone of a good differential diagnosis is a meticulous and comprehensive history of the present illness. Initial evaluation should involve a detailed study of the patient’s anamnesis and pharmacological regimen. Here, the long‐term use of both rivaroxaban and alendronate effectively rules out an acute event such as the one observed. This detailed account of the patient’s symptoms, their onset, and their progression provides vital clues that guide the clinician toward the correct diagnosis. Furthermore, this case highlights the potential risks associated with even routine medical practices. Chlorhexidine, a widely used antiseptic in various clinical settings, is specifically mentioned as a substance that, while generally safe, can induce severe anaphylaxis in susceptible individuals. Anaphylaxis is a life‐threatening allergic reaction that can rapidly progress, affecting multiple body systems and potentially leading to shock and death. This underscores the need for clinicians to be vigilant and to consider even common substances as potential triggers for serious adverse events. Therefore, a complete medical history should include not only a detailed account of the current problem but also a thorough review of the patient’s allergies and past reactions to medications and other substances.

## Funding

No funding was received for this study. Open access publishing facilitated by Universita degli Studi di Trieste, as part of the Wiley ‐ CRUI‐CARE agreement.

## Consent

All patients allowed personal data processing, and informed consent was obtained from all individual participants included in the study.

## Conflicts of Interest

The authors declare no conflicts of interest.

## Data Availability

The data that support the findings of this study are available from the corresponding author upon reasonable request.
